# Esthetic Reconstruction of Diastema with Adhesive Tooth-Colored Restorations and Hyaluronic Acid Fillers

**DOI:** 10.1155/2017/5670582

**Published:** 2017-03-12

**Authors:** Supawadee Naorungroj

**Affiliations:** Department of Conservative Dentistry, Faculty of Dentistry, Prince of Songkla University, Hat Yai, Songkhla, Thailand

## Abstract

*Objective*. This report presents a comprehensive esthetic treatment with adhesive tooth-colored restorations in a combination with hyaluronic acid (HA) fillers of diastema in an orthodontic patient with relapse.* Case Report*. A 36-year-old female patient consulted about 1.5–2 mm midline diastema after an orthodontic relapse of replacing missing central incisors with lateral incisors and dark-colored gingival tissue as a result of a metal post and core with porcelain fused to a metal (PFM) crown at the left lateral incisor. Restorative treatments included replacing the PFM with all-ceramic material and placing a ceramic veneer on the right lateral incisor. To close the space, crown forms of both lateral incisors were altered. A direct resin composite was then used to reform right and left canines to a more ideal lateral incisor shape. An HA fillers injection was used to fill the remaining open gingival embrasure. Eighteen months after treatment, the interdental papilla remained stable and the patient was satisfied with the result.* Conclusion*. Esthetic reconstruction of diastema and open gingival embrasure in this case can be accomplished without orthodontic retreatment. Tooth-colored restorations and HA filler injection appear as a promising modality to address this patient's esthetic concern.

## 1. Introduction

The presence of a space between anterior teeth or diastema is a common esthetic problem in adults. It can be due to a number of reasons including growth and development deficiencies, tooth-sized discrepancies, improper tooth angulations, missing anterior teeth, and pathologic conditions, as well as orthodontic relapse [1–3]. A careful diagnosis and multidisciplinary approach are required to achieve a satisfactory final esthetic outcome for both “white” and “pink” esthetics of treating diastema. “White esthetics” refers to a natural appearance of final restorations with suitable materials, while “pink esthetics” refers to the soft tissue aspects of a tooth including interdental papilla and gingival contour [3–5].

For patients with missing central incisors, an orthodontic procedure is often a treatment option if patients also have increased overjet, marked crowding, or malocclusion of posterior teeth. After the adjacent teeth are migrated into the correct position with acceptable angulation, esthetic reconstruction frequently involves reshaping or altering the substituted teeth with restorations to obtain the proper shape, size, and function [6, 7]. More importantly, a successful treatment requires patient cooperation to wear a long-term retainer to prevent orthodontic relapse. In case of such relapse after an orthodontic correction, there are two solutions for the presence of spacing. The first is an orthodontic retreatment, while the second employs a restorative procedure to close the space. In some cases, although “white esthetics” can be achieved, “pink esthetics,” or reconstruction of the lost interdental papilla, is a problem, especially when the distance from the base of the contact point to the alveolar bone is greater than 5 mm [8, 9]. Recently, the use of an injectable hyaluronic acid (HA) gel for interdental papilla reconstruction has been reported in several clinical studies [10–12]. The results suggest promising levels of patient satisfaction as the improvements in papilla fill can be maintained for periods of 6 months to 2 years [10, 11, 13]. This approach is less invasive when compared to previous sophisticated surgical techniques [4, 14].

This report presents the case of a 36-year-old female with a midline diastema resulting from an orthodontic relapse after lateral incisors were substituted for missing central incisors. The treatments involved esthetic restorative constructions and HA injection without an orthodontic retreatment.

## 2. Case Report

### 2.1. Dental History and Examination

**Figure 1:**
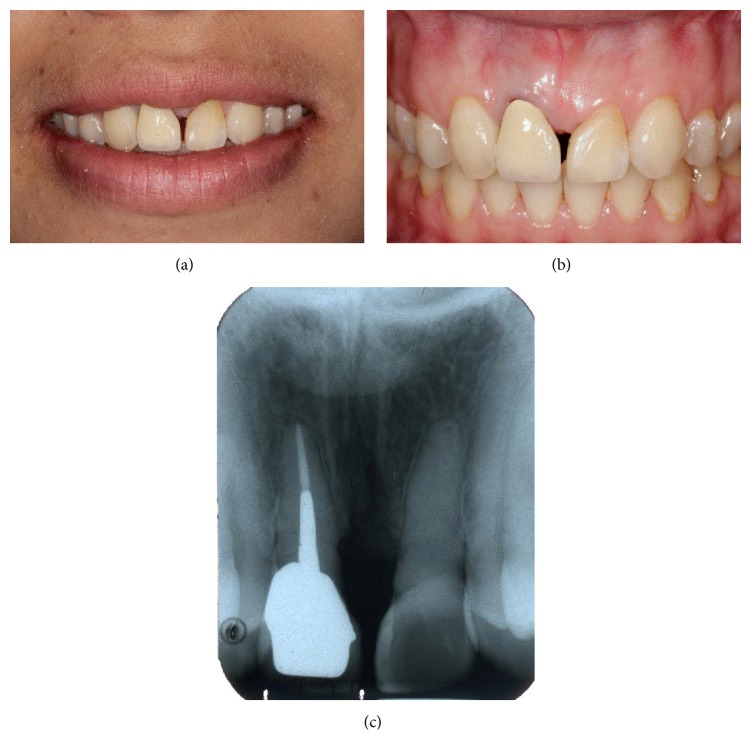
Pretreatment extra and intraoral photograph and periapical radiograph. (a) Smile photograph. (b) Intraoral photograph (front view) showing a midline diastema. (c) Periapical radiograph of the lateral incisors.

**Figure 2:**
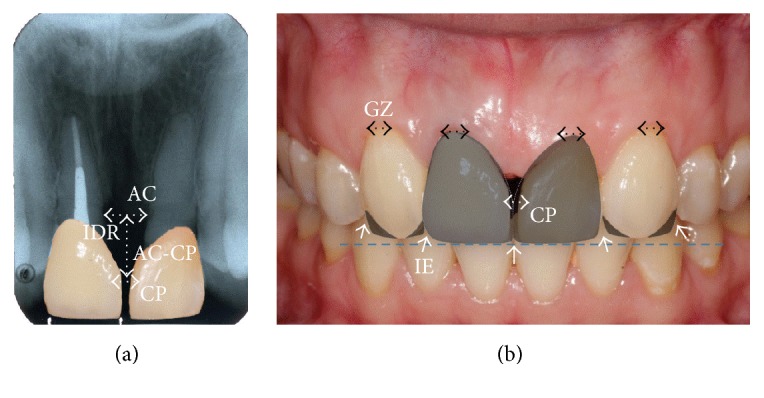
Analysis of “pink” and “white” esthetics and the restorative plan. (a) Radiographic analysis. IDR, the interproximal distance between roots; AC-CP, the distance between the most coronal part of the alveolar bone crest (AC) and the most apical part of the contact point (CP). (b) Clinical photograph analysis. Gingival zenith (GZ) positions, level of upper incisal edge (blue line), incisal edge embrasure (IE), and deficient interdental papilla (black triangle between two teeth) after placement of adhesive restorations are shown. Hyaluronic acid filler injection is planned to fill the deficient interdental papilla.

A 36-year-old female visited the Postgraduate and Specialized Conservative Dentistry Clinic of the Dental School, Prince of Songkla University, due to concern about a space between her front teeth (Figure 1(a)). The patient presented clinically with a midline diastema of 1.5–2 mm. She reported that she had lost two maxillary central incisors due to trauma when she was a teenager. Ten years later, she received orthodontic treatment that moved the lateral incisors into the central incisor positions and the canines into the lateral incisor positions. The right lateral incisor also received an endodontic treatment followed by a casting post and core with porcelain fused to a metal crown (PFM). The left lateral incisor was built up with a direct resin composite (Figures 1(b) and 1(c)). The mesiodistal width dimension of left and right lateral incisors was increased by 1 mm each in order to transform the lateral incisors into maxillary incisors. Gingival recession of about 0.5 mm was presented at the distolabial aspect of the left lateral incisor, resulting in gingival level disharmony (Figure 1(b)). A shine-through effect in a metal ceramic crown and cast metal post was also observed (Figure 1(b)). Orthodontic treatment and all restorations were completed in a private clinic more than 13 years ago. A periapical radiograph showed that both lateral incisors had horizontal bone loss at cervical 1/3 of the roots. The distance from the estimated contact point to the alveolar bone was about 8 mm (Figures 1(c) and 2(a)). This suggested that it was unlikely that a restorative technique alone would completely close the space.

### 2.2. Treatment Objective and Plan

The primary treatment objective was to restore a normal appearance of both “white” and “pink” esthetics to the maxillary anterior teeth. Based on the objective and given the clinical and radiographic features, two treatment options were proposed to the patient. The first option consisted of an orthodontic retreatment for diastema closure and papilla reconstruction with a surgical technique for correction of the open gingival embrasure. With orthodontic tooth movement, appropriate crown-root angulation, incisal level, and gingival zenith position for final restorations can be achieved. However, there is a longer time and higher cost of treatment compared to the second option. In addition, an outcome of papilla reconstruction is unpredictable. The second option consisted of the esthetic reconstruction of the anterior teeth without orthodontic movement by increasing the mesial emergence profile and the additional mesiodistal width of the lateral incisors (0.75 mm each) with HA injections to treat the interdental papilla loss. To correct the gingival level disharmony of the two lateral incisors, gingival sculpting of the left lateral incisor with a diode laser was carried out. This option is less invasive, fast, and low-cost solution to treat the midline diastema and the open gingival embrasure. However, HA retreatment may be required in long term. For both options, all-ceramic crown and veneer were used for esthetic reconstructions of the lateral incisor substitution for the missing central incisors. Esthetic reshaping of canines with direct resin composite build-ups would be substituted for the lateral incisors. As far as the esthetic outcome was concerned, the risks and benefits of replacing the metal post with an esthetic post were discussed with the patient; however, she declined the metal post replacement and decided on the second option.

### 2.3. Treatment

A study model and a diagnostic wax-up were used for esthetic evaluation and fabricating provisional restorations. In addition, the diagnostic wax-up and photograph were used to facilitate communication with the patient, periodontist, and laboratory technician.  Figure 2(b) shows a final restorative plan including the all-ceramic crown for the right lateral incisor, the ceramic veneer for the left lateral incisor, and the direct resin composite build-up of both canines. Injection of HA fillers was planned to fill the deficient interdental papilla, because there was an excessive distance from the alveolar bone crest to the approximal contact in this case.

**Figure 3:**
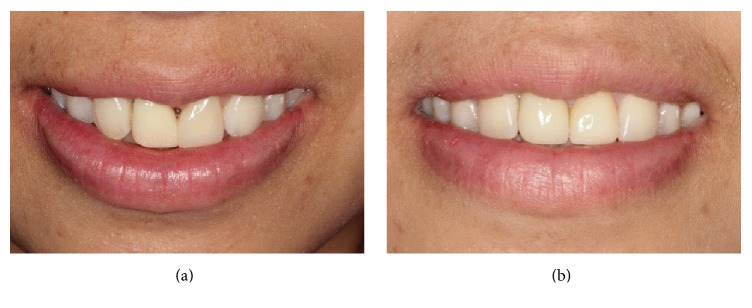
Front views of intraoral photographs of the patient's smile. (a) After placement of provisional restorations. (b) At 2 weeks after completing treatment.

In the first treatment visit, after removal of the PFM crown and previous resin composite restoration, a provisional crown made with Protemp™ (3M ESPE) and a provisional veneer made with direct resin composite (Filtek Z350 XT, shade A2B, 3M ESPE) with a spot etching technique were placed (Figure 3(a)). At this point, the mesiodistal width of the lateral incisors had increased a total of 1.75 mm each. The tip of the interdental papilla was blunt and lay between the interdental contact points at the labial cementoenamel junction (CEJ) level. There was about 1.5–2 mm of the open gingival embrasure remaining between teeth, measured from the tip of the interdental papilla to the contact point (Figure 3(b)).

**Figure 4:**
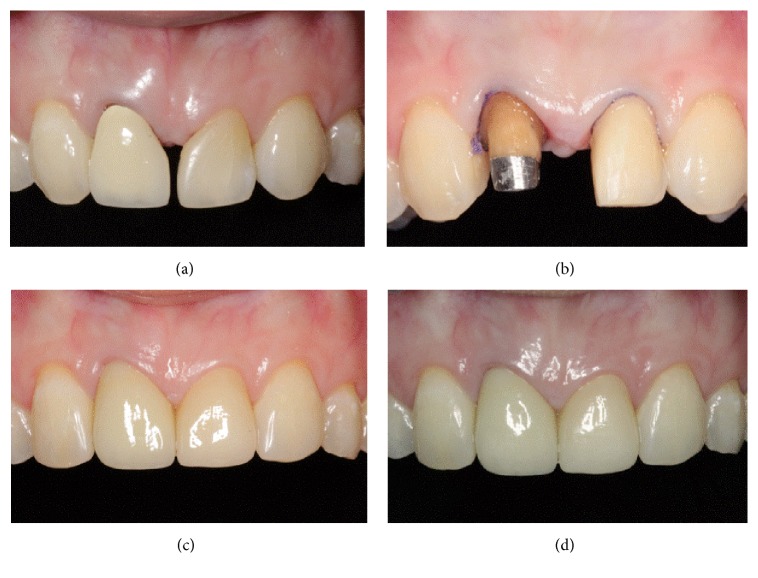
Front views of intraoral photographs. (a) Before treatment. (b) Teeth preparation. (c) At 2 weeks after completing treatment. (d) At 18 months after completing treatment.

In the following visit, a gingivectomy with a diode laser of the left lateral incisor and direct injection of HA (JUVÉDERM® Ultra XC) of 0.2 cc into the middle of the papilla using a 30-gauge needle were done under a topical anesthetic (10% xylocaine). The HA injection was repeated at 21 days with a dose of 0.2 cc and then at 48 days with a dose of 0.05 cc. Gentle massage of the treatment area for about 1 minute was done. After each treatment session, the patient was given postoperative instructions that included using a soft toothbrush and 24-hour abstinence from mechanical plaque control in the treatment area [10]. The patient reported postoperative discomfort experienced at the last time of injection. At a 1-month follow-up after the last injection, about 1 mm open gingival embrasure or black triangle space in the cervical region remained. Subsequently, the proximal contours of the provisional crown and veneer were added with a flowable resin composite (Filtek Z350 XT flowable, 3M ESPE) and the contact area was lengthened and located more apically to close the remaining open gingival embrasure. One month later, final tooth preparation and impressions were done. The material used for the crown of the right lateral incisor was zirconia with IPS E-max veneering, while the veneer of the left lateral incisor was IPS E-max. After checking for suitability, the all-ceramic crown was fixed with RelyX Unicem (3M ESPE) and the veneer was fixed with RelyX veneer shade WO (3M ESPE). During the same visit, the reshaping and direct resin composite build-up of the canines (Filtek Z350 XT shade A3E, 3M ESPE) to substitute for the lateral incisors were completed (Figure 3(b)). The esthetic appearances of the restorations and soft tissue were reevaluated at 2 weeks, 3 months, 6 months, 12 months, and 18 months after completing treatment. Routine scaling and polishing were done during the 12-month follow-up.  Figure 4 shows intraoral photograph of adhesive tooth-colored restorations and the interdental papilla from baseline, 2 weeks, and 18 months after completing treatment. The length of lateral incisors that were substituted for missing central incisors was about 0.5 mm longer than incisal edges of the adjacent teeth and the interdental embrasures were shaped as inverted “V.” Gingival contours of the restored anterior teeth were not ideal as they were at the same height. However, gingival contours of the restored anterior teeth were considered as acceptable, provided there was no gingival margin exposure at smiling. The overall final clinical results of restoring the maxillary anterior teeth in this patient were satisfactory, even though the esthetic outcomes were compromised by a shine-through effect from the metal post and compromised gingival contour.

## 3. Discussion

This case report demonstrated that the patient's esthetic appearance and smile were improved with adhesive tooth-colored restorations and an HA fillers injection. The esthetic reconstruction in this case was challenging due to the absence of maxillary central incisors, divergent root angulation, increased distance between alveolar bone-interproximal contacts of substitute teeth (lateral incisors), triangular crown morphology, discrepancy of gingival level, and thin periodontal biotype.

In the event of missing maxillary central incisors, the straightforward option is to arrange the space for implant placement of the missing incisors. The alternative option is lateral incisor and canine substitutions by orthodontic movement followed by esthetic reconstruction [7]. However, if a patient fails to wear retainers, orthodontic relapse often occurs. This patient presented at our clinic as concerned about anterior spacing after orthodontic treatment, which is a common complaint in adults seeking esthetic dental treatment. A web-based survey regarding esthetic perception among lay people reported that anterior diastema and midline deviation received the worst ratings [15]. Another survey study found that open gingival embrasure, or black triangle, greater than 3 mm was perceived as noticeably unattractive by both general dentists and lay people, while orthodontists rated a 2 mm open gingival embrasure as less attractive than an ideal smile with healthy gingival embrasure [16].

The ultimate goal in treating this patient is to close the midline diastema without creating an open gingival embrasure. The absence of interdental papilla can cause plaque accumulation, phonetic problems, and esthetic deformities. In addition, open gingival embrasure can affect a patient's smile [4]. Therefore, preservation or reproduction of the interdental papilla in the gingival embrasure of the esthetic zone should be considered in restorative, orthodontic, and periodontal treatments [3, 14, 17]. As previously mentioned, this particular patient had several limitations and risk factors contributing to anterior spacing and lost interdental papilla. When formulating a treatment plan, these parameters need to be carefully considered to establish the balance between teeth (e.g., size, shape, contour, and contact area) and gingival architectures (e.g., intact papilla and symmetrical gingival contours). With orthodontic retreatment, the lateral incisors will be moved closer and their roots made parallel to reduce the likelihood and severity of open embrasure. Divergent roots are found to be strongly associated with open gingival embrasures. The contact point will lengthen and locate more apically toward the papilla when root angulation becomes increasingly parallel [14, 18]. In addition, the lateral incisors may be intruded and the canines may be extruded to create the natural gingival level. However, the orthodontic treatment is a more complex, longer, and expensive treatment compared to restorative treatments, all of which were the major concerns of this patient.

As the patient had time and financial constraints along with a decision not to disturb a good occlusion with an acceptable profile, it was decided that the esthetic reconstruction with adhesive tooth-colored restorations (crown, veneer, and resin composite build-up) and HA gel injection to treat midline diastema and interdental papilla loss without orthodontic intervention was an appropriate treatment option for this patient. It was unlikely in this case that the embrasure space could be completely filled by the gingiva even after altering the crown morphology or increasing the emergence profile of the restorations. From the assessment, the distance between the contact point and the alveolar bone crest was about 8 mm. After placement of the restorations that altered the mesiocervical areas, there would be a ~1.5–2 mm black triangular space remaining. A classic study by Tarnow et al. suggested that when the distance was 5 mm, the papilla was present in almost every case. When the distance was 6 mm and 7 mm, the papilla only appeared in approximately half (56%) and one-fourth (27%) of the cases, respectively [8]. Several surgical techniques were introduced to reconstruct the lost interdental papilla. One key factor for a successful surgical approach is that the patients have a thick gingival biotype as it has a better vascular supply and patients tended to maintain the papilla height. Currently, however, the uncertainty of surgical procedures to reproduce interdental papilla has been acknowledged. Surgical procedures may lead to contraction and necrosis of grafted tissue because of low blood supply and tissue fragility [4, 14]. Such approach was, therefore, inappropriate for this patient. Another problem related to the thin gingival biotype in this case is the shine-through effect of the cast metal post.

An HA gel injection, a nonsurgical treatment, is a more conservative approach. HA gel has been used as a dermal filler and was recently used to reconstruct lost interdental papilla [10]. HA is a polysaccharide (glycosaminoglycan) molecule found in body tissues including periodontal tissues. When in a gel form, it binds to water and swells, resulting in smoother/fuller tissue contours [10, 19]. Several clinical studies have shown that the HA injection is safe and effective as there is an improvement in the papillary space and the patient's smile. Postoperative discomfort after injection was the main complaint reported by the patients; however, most of those patients opted to undergo the treatment procedure again [10]. For a long-term assessment, one study has shown that there was an improvement at 6 months and that the interdental papilla could be maintained for up to 2 years [11], while another study indicated that there was a slight relapse between 4 and 6 months [10].

In summary, the esthetic rehabilitation of patients presenting with orthodontic relapse in anterior maxillary teeth substitution is challenging because of the need for a multidisciplinary approach. Additional deficiencies present in conjunction with spacing, such as soft tissue and bone defects or the existence of improper restorations, must be considered. In addition, a careful analysis of the relationship between restorations and gingiva must be made before treatment. By taking such considerations into account, the long-term stability of diastema closure with adhesive tooth-colored restoration and reconstruction of interdental papilla in the esthetic zone can be achieved.
